# Investigation of Product and Process Fingerprints for Fast Quality Assurance in Injection Molding of Micro-Structured Components

**DOI:** 10.3390/mi9120661

**Published:** 2018-12-15

**Authors:** Nikolaos Giannekas, Per Magnus Kristiansen, Yang Zhang, Guido Tosello

**Affiliations:** 1 Department of Mechanical Engineering, Technical University of Denmark, Produktionstorvet, Building 427A, DK-2800 Kgs. Lyngby, Denmark; yazh@mek.dtu.dk (Y.Z.); guto@mek.dtu.dk (G.T.); 2 Institute of Polymer Nanotechnology (INKA), FHNW University of Applied Sciences and Arts Northwestern Switzerland, School of Engineering, Klosterzelgstrasse 2, CH-5210 Windisch, Switzerland; magnus.kristiansen@fhnw.ch; 3 Laboratory for Micro- and Nanotechnology, Paul Scherrer Institute, CH-5232 Villigen-PSI, Switzerland

**Keywords:** precision injection molding, quality control, process monitoring, product fingerprint, process fingerprint

## Abstract

Injection molding is increasingly gaining favor in the manufacturing of polymer components since it can ensure a cost-efficient production with short cycle times. To ensure the quality of the finished parts and the stability of the process, it is essential to perform frequent metrological inspections. In contrast to the short cycle time of injection molding itself, a metrological quality control can require a significant amount of time and the late detection of a problem may then result in increased wastage. This paper presents an alternative approach to process monitoring and the quality control of injection molded parts with the concept of “Product and Process Fingerprints” that use direct and indirect quality indicators extracted from part quality data in-mold and machine processed data. The proposed approach is based on the concept of product and process fingerprints in the form of calculated indices that are correlated to the quality of the molded parts. A statistically designed set of experiments was undertaken to map the experimental space and quantify the replication of micro-features depending on their position and on combinations of processing parameters with their main effects to discover to what extent the effects of process variation were dependent on feature shape, size, and position. The results show that a number of product and process fingerprints correlate well with the quality of the micro features of the manufactured part depending on their geometry and location and can be used as indirect indicators of part quality. The concept can, thus, support the creation of a rapid quality monitoring system that has the potential to decrease the use of off-line, time-consuming, and detailed metrology for part approval and can thus act as an early warning system during manufacturing.

## 1. Introduction

In recent years, market and consumer needs have led to a shift in the design of complex products by focusing on product design for high volume, cost-effective manufacturing processes in many applications such as automotive components, communication, and medical devices particularly in micro-sized applications [1,2]. Injection molding is one of the processes that can ensure a cost efficient production with short cycle times. Therefore, it has been the favorite process for many manufacturers of cost-effective products and reportedly now accounts for 50% of the produced plastic parts [3].

In many sectors and especially in the medical field, numerous products have micro features with tight tolerances. Satisfying the product specifications and functional requirements for all injection-molded components is a difficult task and requires a highly stable process. Performing frequent metrological inspections is essential to ensure process stability and produced part quality assurance and to approve a production batch. However, metrological inspections require a great deal of resources and effort in comparison to the cost-effectiveness and short cycle time of the injection molding process. Due to the demand for tighter tolerances especially in micro parts and parts with micro features, process monitoring has been the object of many research efforts. The ultimate objective is the monitoring of an optimized process to reveal the occurrence of defects and to ensure that the process produces parts within the specification limits. Out-of-tolerance production can result in significantly increased production costs and a high scrap rate, which reduces the efficiency of the process especially in industrial situations where the components are left to rest for up to 48 h prior to a metrological inspection.

The current paper presents an innovative approach to part quality monitoring and control by proposing indices that serve as part quality indicators (QI) and as “Product and Process Fingerprints” that are based on both process and product data. The proposed approach takes two parallel tracks as follows.

First, the “Product fingerprint” track in which the use of micro-features positioned on the molded part and their replication quality is considered to be directly connected to the overall quality of the component. The correlation of the replication fidelity of these microfeatures on the runner with those of the part itself is explored. Recent research studies have provided examples of part features used for the correlation with part quality. Examples can be found in the use of the weld line positions to assess the quality of the molded part, as described by Tosello et al. [4], and the use of nano features placed on different areas of a component that provides the necessary indicators for rapid part quality assessment as discussed by Calaon et al. [5].

Second, the “Process fingerprint” track explores the use of transient process data that originates from the in-mold sensors and the machine control sensors together with data from the on board quality system of the injection molding machine for process monitoring. Multiple research studies have been conducted with different approaches in the field of sensor technology as a tool for process control and optimization in an attempt to decrease the intensive metrological inspections required for the approval of injection-molded parts. Some of the studies used in/on-mold sensors to regulate and monitor the process with promising results [6,7,8,9] with increased tooling costs. Mold separation (MS) monitoring with the use of a linear variable differential transformer (LVDT) is one of those techniques and can provided a reliable indicator for part weight and thickness [9]. Gao et al. [6] used a custom designed multivariate sensor (MVS) to monitor the quality of the injection-molded parts assuming that the part quality indicators (dimensions) can be tightly controlled when the in-mold process parameters are known.

Other studies make use of either data from external sensors placed on the molds or data from in-line measuring equipment to record indirect process parameter data in order to optimize the process with respect to the functional requirements. Johnston et al. have utilized an in-line multivariate optimization system for process control and optimization [10]. Yang et al. make use of digital image processing in-line with the process for defect detection purposes [11]. After detection of a defect, the detection algorithm feeds data to an algorithm for process optimization based on a model-free optimization (MFO) procedure.

Scientific research in this field is not limited to the use of in-line experimental process monitoring of conventional injection molding. In fact, the study by Wang et al. presented a work on warpage optimization with a numerical simulation procedure of dynamic injection molding and sequential optimization based on the Kriging surrogate model [3]. Other studies rely on the application of artificial neural networks (ANN) and genetic algorithms for optimization and monitoring of the process [12].

The previously mentioned approaches focus on the case of a tightly controlled and optimized process. However, the dimensional control of the injection-molded components is not considered directly. The resulting part quality is the main objective of any quality assurance system. Thus, coupling of the dimensional accuracy of the parts with the sensor data is required and this is addressed in the present research.

**Figure 1:**
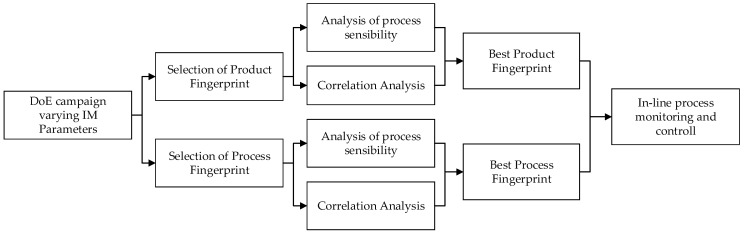
Flow chart for procedure for product and process fingerprint to measurand identification.

The approach presented in the current article is based on process and product fingerprints. The paper in divided into three sections. Section 1 is the Introduction. Section 2 includes the Materials and Methods in which the materials and methods used for the experiment, the test geometries, collected data, and analysis methods are presented. A test geometry with two cavities was fitted with a number of both functional and test micro structures to serve as “product fingerprint” candidates. Section 3 includes the Results and Discussion in which several process variables and signals were collected to extract candidates for “process fingerprints” using the analytical methods presented. The results of the analysis are presented and a discussion is commenced with the “product fingerprint” candidates’ correlation of the test structure to the functional ones to be assessed. Similarly, the “process fingerprints” candidates were subject to correlation analysis with the features measurements to identify the most suitable ones to act as indicators of the overall quality of the part. The discussion proceeds with a comparison of process and product fingerprints so that the most suitable “fingerprints” for an in-line process monitoring and control system may be selected. Setting up such a system requires the coupling of the selected measurand/product quantity/fingerprint to the proper process fingerprint as shown in the procedure schematically outlined in Figure 1. The last section of the paper summarizes the findings and proposes fingerprints and a way to extract them.

## 2. Materials and Methods

### 2.1. Case Study and Geometries in Use

**Figure 2:**
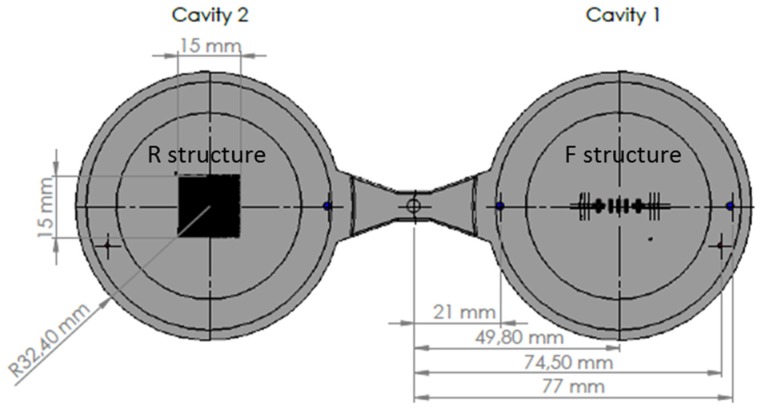
Structures of interest on full molded part. Cavity 1 includes the F structure, which is presented in more detail in Figure 3. Cavity 2 includes the R structure, which is presented in more detail in Figure 4. The F structure is in Cavity1 and the R structure is in Cavity 2.

**Figure 3:**
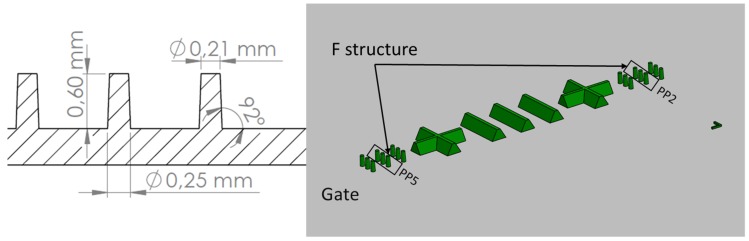
Cavity 1, F structure. Micro pillars used to assess part quality and process fingerprints are marked on the figure. PP5 indicates micro features near the gate while PP2 indicates micro features far from the gate.

**Figure 4:**
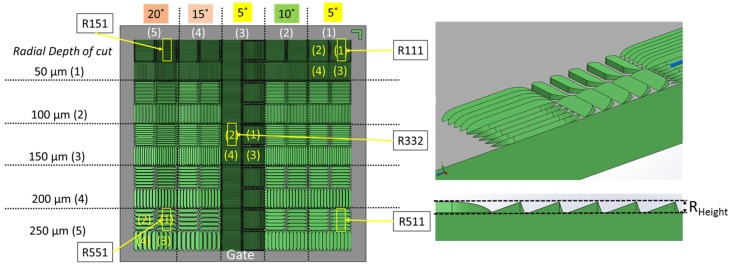
Cavity 2, R structure array created with different radial mill tool engagement (50–250 μm) and inclination angle (5°–20°). Positions of interest used to assess part quality and process fingerprints are marked: R551 and R511 indicate micro features (micro ridges) near the gate on the left and right, respectively. R151 and R111 indicate micro features far from the gate on the left and right, respectively. The position R332 indicates micro features centered in the middle of the part. For this case and for all μ-features of column 3, the inclination and the inclined plane length were kept to 5° and 50 μm, respectively.

The geometry of the molding that was studied was a component disk (see Figure 2) used for testing functional micro-structures and nanostructures. The concept of process and product fingerprint as discussed in the introduction appeared promising but had not yet been tested. In order to examine the viability of the concept, a number of micro-feature geometries were considered as test subjects but only two were selected. Figure 3 and Figure 4 present the two types of micro-features. One consists of different geometries and the measurements were focused mainly on the conical micro pillars positioned in the structure, designated as the “F structure,” and the other consists of micro ridges structures designated the “R structures.” For the purpose of this paper, the micro features in all positions will be stated as “measurands.” It should be noted that the R structures in the particular application are inclined planes created in the mold insert with different angles and mill tool radial engagement, which is shown in Figure 4. The alphanumeric codes for both structures represent the selected measurement positions while Table 1 provides a summary of the characteristics of the micro features and the sensor outputs. More details about the selected structures are provided in Section 2.4 where the measurement strategy for structure characterization is described.

### 2.2. Experimental Setup and Mold Design

**Figure 5:**
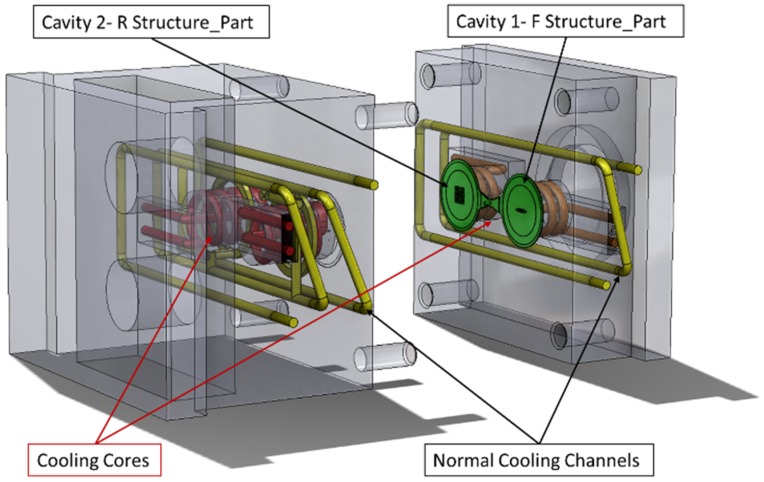
Mold geometry and cooling channels.

**Figure 6:**
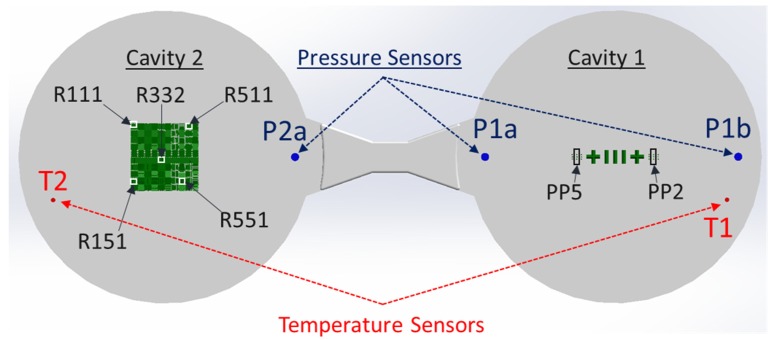
Measurement positions of structures and sensor position on full molding. In this case, the sensor positions are shown in the front side of the molding for easier association of the feature measurement to the sensor recordings. The F structure is in Cavity1 and the R structure is in Cavity 2.

After the geometries of the test micro feature geometries had been selected, two mold inserts were machined to include the features on the two cavities of a test mold (Figure 2, Figure 3, Figure 4, Figure 5 and Figure 6). The inserts were installed on a three-plate mold that was designed to be compatible with both injection and injection compression molding. The plate holding the cavities could slide on secured guideways and the “gap” in the case of ICM was determined by springs at the back side of the plate. For the experiment discussed in this paper, the gap was kept at 0 mm with the springs fully compressed. The ejection of the part was facilitated by the use of four ejector pins at the periphery of the disk part cavity (Ø65 mm), which was larger than the micro-structured area of the mold insert (Ø45 mm).

The mold described above provided in-mold process monitoring capabilities since it contained three N-group thermocouples (4003B) and three piezoelectric pressure sensors (6006BB—Sensitivity of sensors listed in Table 2) from Priamus and was supported by the Priamus Fillcontroll system for in-mold flow front monitoring and process control. Additional thermocouple sensors were positioned in the mold to control the variothermal IM processes. However, this study was conducted under isothermal process conditions. The positions of the sensors in the mold are illustrated in Figure 6.

### 2.3. Experiment Details

**Figure 7:**
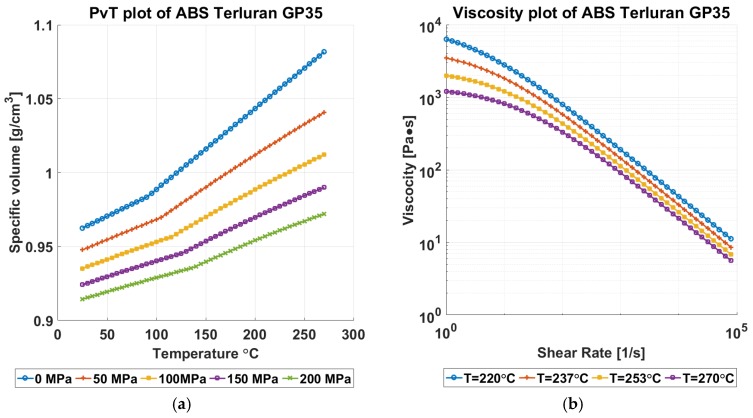
(**a**) PvT and (**b**) viscosity plots of material Styrolution Terluran GP-35 (ABS) [18].

A full 2^4^ × 3 full factorial designed experiment was conducted to examine the validity of the proposed product and process the fingerprint concept. The process parameter of melt temperature “*Tm*” (°C), mold temperature “*Tmld*” (°C), injection speed “*InjSp*” (mm/s), and packing pressure “*PackPr*” (bar) were used in the experiment in which it is proven through well-established research [13,14,15,16,17] that these are the most significant process parameters that affect the part quality in injection molding. The levels of the parameters were set with respect to the process parameter ranges indicated in the material datasheet. For this study, a commercial grade of Acrylonitrile Butadiene Styrene (ABS) (Styrolution Terluran GP-35), which was characterized by a relatively large processing window was used. The limits of the window were used to set the experimental parameter levels, as shown in Table 3, in order to map the effect of processing conditions on the replication of the micro pillars of the F structure and the micro ridges of the R structure. The experiment was performed on an Arburg 320A-Allrounder 600-170 injection-molding machine (Arburg GmbH +Co KG, Lossburg, Germany) with a clamping unit of maximum 600 kN clamping force and a screw diameter of 30 mm. The melt temperature profile was set by using 5 °C intervals in each heating zone of the reciprocating screw to facilitate a gradual heating of the material throughout the barrel. The packing (t_pack_ = 8s) and cooling (t_cool_ = 20s) times were set as values that were high enough to avoid any influence on the experimental results. Figure 7 shows the PvT (7a) and the viscosity (7b) diagrams of the material used in the experiment.

Through the use of a DOE approach, two objectives can be met. First, proving that the structures selected were sensitive to the process variation and could be correlated to the part quality, they could be used for quality monitoring as suitable product fingerprints. Second, the data originating from the experiment could be used for the determination of the process fingerprints required to run an in-line process monitoring system as the quality indicators with better correlation with product quality.

### 2.4. Measurement Strategy and Procedure

From the experiment and for every experimental treatment, the initial 20 molded parts from the start of the process were discarded since the process had not reached stability. The next 10 parts were then collected for assessment with three parts to be measured for the assessment of micro feature replication quality on the parts (μ-pillars (F-structure) in Cavity 1 and μ-ridges (R-Structure) in Cavity 2). It was decided to use the initial, middle, and last part of the collected sample (parts 21, 25, and 29).

As shown in Figure 3, Figure 4 and Figure 6, two positions in Cavity 1 (micro pillars/F structures) and five positions in Cavity 2 (micro ridges/R structures) were selected as possible candidates for product fingerprints. In particular, the five positions were selected with respect to the limits of the inclination and the inclined plane length ranges in order to access the replication fidelity in the two size limits of the R structure. It is important to note that the μ-pillar structures in Cavity 1 were designed as functional microstructures on a microfluidic biochip system, as reported by Marhöfer et al. [19]. However, the structures in Cavity 2 due to their smaller size could be incorporated into the produced component and could easily act as a product fingerprint. In the present case, the two different types of structures were located in different cavities. Since they were symmetrically opposed and balanced, it can be readily assumed that the molding conditions in the two cavities are similar. In all cases for the assessment of the feature quality, the height of the feature was set as the measure and will be denoted by the measurand’s name.

Due to the differences in structure type and size, the measurement strategy for the two types of microstructures was not identical but was instead optimized for each case separately, which kept similar setting levels when possible. The feature height dimensional measurements were carried out with the use of a 3D confocal laser scanning microscope (CLSM, Keyence VK-X210 from KEYENCE, Osaka, Japan).

#### 2.4.1. Pillar Dimensional Measurement and Error Evaluation Procedure

Even though the measuring instrument had been designed for the measurement of complex structures, acquiring a full scan of the pillars is still a challenge due to the almost vertical slopes (92°, see Figure 3) of the pillars. Table 4 includes the settings of the microscope that were used for the measurement of the micro-pillars of the F structure.

**Figure 8:**
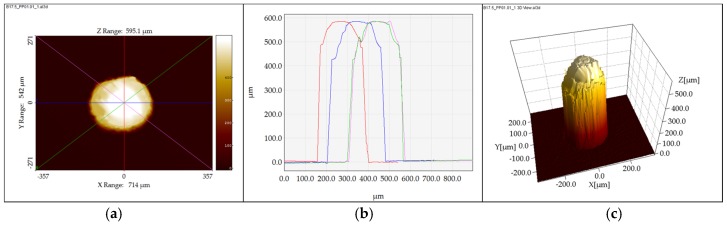
Pillar height measurement [20], (**a**) the four planes intersecting the middle of the pillar for the extraction of the cross-section profiles, (**b**) the four cross-section profiles of the pillar, and (**c**) the 3D representation of a pillar.

In order to assess the stability of the process, the effect of the process parameter changes, and the replication fidelity of the pillar micro features per experimental run, three pillars in each position of interest were scanned to measure the pillar height. The middle μ-pillars in Positions C1PP2 and C1PP5 (Figure 8) were measured five times in order to ensure the repeatability of the measurements (standard deviation in the range of μm) and provided sufficient data for error estimation. The measurement files were consequently processed with the use of SPIP 6.4.1 (SPIP^TM^, Image Metrology A/S, Hørsholm, Denmark) software to extract four 2D pillar profiles from each scan.

The average pillar height was calculated by using four profiles (see Figure 8b) that intersected the center of the pillars. The procedure used scans of both the mold and the molded parts to calculate a measure of the replication fidelity of the molding process.

#### 2.4.2. Procedure for Dimensional Measurements of the R Structures

**Figure 9:**
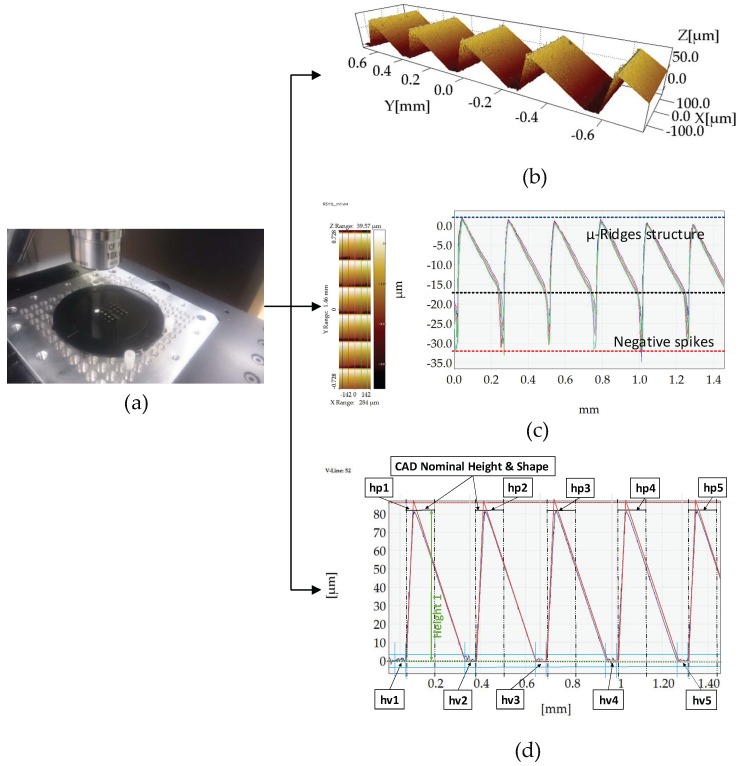
(**a**) Measurement of the R structure on a Keyence CLSM microscope. (**b**) SPIP 3D scan of position C2R551. (**c**) Cross-section profile extraction. In this scenario, the position C2R511 is used to portray the negative “spikes” caused by the replication of the burrs. (**d**) Illustration of a measurement procedure example for peak height (R_height_) on the profiles of position C2R551.

As for the parts produced in Cavity 1, μ-ridges (R structure) on parts produced in Cavity 2 were measured with the use of the same Keyence confocal laser microscope (Figure 9). The CLSM microscope settings are given in Table 4. Similarly to the μ-pillars of Cavity 1, in order to assess the stability of the process, the effect of the process parameter changes and the replication fidelity of the micro-ridges (R structure) as a measuring procedure was devised. Five positions for each part in each experimental run were scanned to access the average area feature height. The measurement positions are illustrated in Figure 4. The measurement files were consequently processed with the use of SPIP 6.4.1 software to extract 2D profiles from the scans of each area.

**Figure 10:**
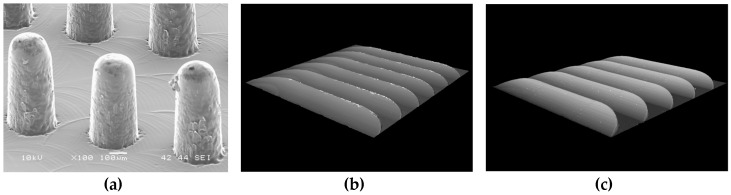
3D images (**a**) micro pillar (F structure) and micro ridges (R structure) features in positions (**b**) C2R511 with 5° inclination and (**c**) C2R551 with 20° inclination.

In Figure 9b, it is important to note the existence of negative “spikes” in the valleys of the surface profile. These surface outliers are not artefacts caused by the laser measurement method. They are physical artefacts that were produced as the negative of the mold insert. The milling strategy on the surfaces of the mold insert that were milled with an angle of 5° produced a large percentage of burr-covered features and consequently the burrs were replicated as deep valleys on the surface of the parts (Figure 9). 3D images of the injection molded micro features provided in Figure 10 illustrate the quality of the features.

Figure 9d shows the measurement procedure used in the Matlab measurement script in order to calculate the average feature height in each area based on the five single profiles extracted from each scan. The profiles are then processed in a Matlab script where the profiles are corrected for tilt and cross-correlated for alignment to the reference profile from the CAD file of the surface. The mean value of the points at the valleys (in specified regions) is calculated (hv1, hv2, hv3, hv4, hv5, ..., hvn) for all five profiles and the point with the maximum value at the region of the peaks is identified (hp1, hp2, hp3, hp4, hp5, …, hpn) (Figure 9d). To counterbalance the effects of the burrs on the profile and to acquire reliable measurements, the five measurements per peak in each position were subjected to an outlier removal algorithm that used the modified IQR criterion (Equation (1)) [21] to remove the outlier values caused by the burrs. The burr-filtered data were then used to calculate the height of the feature (i.e., Height1 = hp1-|hv1|). Once the height of each peak is calculated, the Chauvenet criterion [21] (Equation (2)) was applied to the five values per peak (five profiles) so that the average height of each peak on the profiles and the average area height could be calculated for all measured parts in each experimental run (Figure 9).

(1) Modified IQR-RangeMod.IQR=1.5 IQR∗[1+0.1∗ln(n10)]where: IQR=3rdQuartile−1stQuartile, of the data    n:sample size

(2) $$\begin{array}{l} \mathrm{Chauvenet}\;\mathrm{Criterion}\\ P_{t}=2nP_{xL}=\frac{1}{2}\\ where\;P_{t}:\mathrm{probability}\;\mathrm{band}\;\mathrm{centered}\;\mathrm{on}\;\mathrm{the}\;\mathrm{sample}\;\mathrm{mean}\\ \;n:sample\;size\\ \;P_{xL}=1/\left(4n\right)\;:\mathrm{probability}\;\mathrm{represented}\;\mathrm{by}\;\mathrm{one}\;\mathrm{tail}\;\mathrm{of}\;\mathrm{the}\;\mathrm{normal}\;\mathrm{distribution} \end{array}$$

### 2.5. Process Monitoring and Data Collected

In addition to the physical part measurements, a number of process variables were recorded from the injection molding machine and from external sensors. The first type of data (Machine Data) is routinely exported from the injection molding machine’s controller and is the easiest to access. The data are used for the quality monitoring subroutine of the machine and can be viewed from the machine monitoring software. The *second type of data* (Machine Signals) is the signals from the sensors used to control the process and the machine. In particular, the injection pressure signal is monitored via a strain gauge transducer positioned at the nozzle of the screw. Injection speed and the screw position are monitored via a linear position sensor. The third type of data (In-Mold Sensors Signals) were recorded with the use of in-mold temperature and piezoelectric pressure sensors with a sampling frequency of 250 Hz (see Section 2.2). A full list of the recorded variables is given in Table 5. The signal data from both the IM machine controller and the Fillcontroll system were recorded separately and were aligned with respect to the time scale since the Fillcontroll system has a maximum delay of 4 ms from the incidence of injection until the start of the recording.

**Figure 11:**
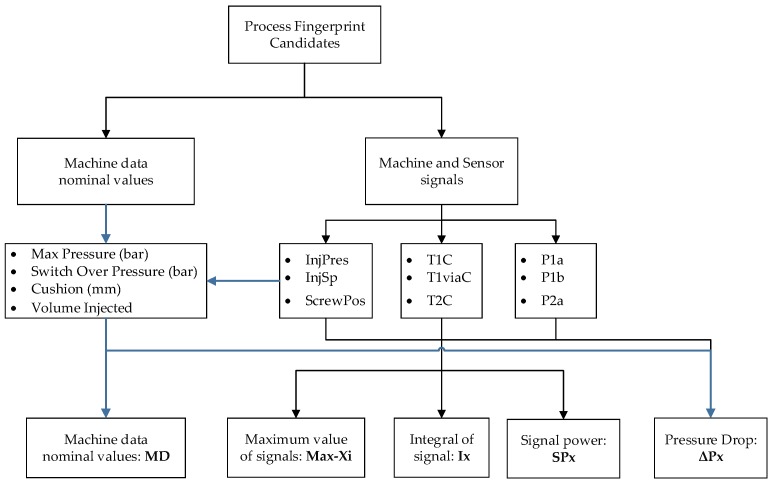
Procedure for the extraction of process fingerprint candidates from the collected data.

Before an investigation of the “fingerprint” candidates, an initial analysis of the recorded signals found no unexpected results such as spikes or delays in pressure and temperature signals from the machine and in-mold sensors. Considering the large number of recorded variables and acquired data, many different types of quality indicators were considered as viable process fingerprint candidates and their correlation to the IM parameters was studied. The different types of process fingerprint candidates can be categorized as single value indicators for each molding cycle, which was illustrated in Figure 11.

In particular, the shape and amplitude of the recorded values and signals were assessed for their viability as process fingerprint candidates. They are explained below.

- **MD**: Machine data nominal values as listed in Figure 11 are single value quantities that originate from the machine controller’s quality control subroutine that records quantities during critical points in the process. Such quantities are: the *Max Pressure* (*MaxPr*) in the nozzle occurring for both injection and packing phases, the switch over pressure, and the cushion left in the barrel after the packing phase of the process has been completed and can provide an indication of the filling deviation from cycle to cycle. In addition, cushion and the maximum screw position (*MaxScPos*) are combined to calculate the non-compressed material volume injected (*VolInj*) into the cavity.
- **Max-Xi**: Maximum values from the recorded signals *Xi* originated from the machine controller (*InjPr, InjSp, ScPos*) and in-mold temperature and pressure sensors.
- **Ix**: is the integral of the whole signal *y(t)* recorded from the start of the injection phase (t_0_ = 0 s) until the end of the packing phase (t_n_ = 8 s), as seen in Equation 3. The integral directly relates to the energy stored in the polymer and can differ from the measured quantity. In particular, the integral calculated from the pressure signals represents the energy stored in the polymer from the melting, compression, and injection of the polymer to the mold cavity.
  (3) $$I_{x}=\int_{0}^{T}y\left(t\right)dt\;where:\;T=end\;time\;of\;signal\;duration\;\left(time=8s\right)$$
- **SP-X_i_**: The power of a signal *X_i_* is the sum of the absolute squares of its time-domain samples divided by the signal length or the square of its RMS level. As for the integral of a signal, the power of the signal relates to the energy of the system for all recorded signal frequencies.
  (4) $${\mathrm{SP}}_{x}=\underset{T\to \infty }{\lim}\frac{1}{T}\int_{0}^{T}{\left|y\left(t\right)\right|}^{2}dt\;where:\;T=end\;time\;of\;signal\;duration\;\left(time=8s\right)$$
- **ΔP-X_i_**: refers to the pressure drop from the pressure at the nozzle of the IM machine to the position of the sensor in the mold for both injection phases, which shows the pressure drop at the gate of the cavity *ΔP-P1a* at the switch over point *ΔP-P1b* and during the packing phase *ΔP-PackP1b*.

## 3. Results and Discussion

### 3.1. Product Fingerprint Analysis

**Figure 12:**
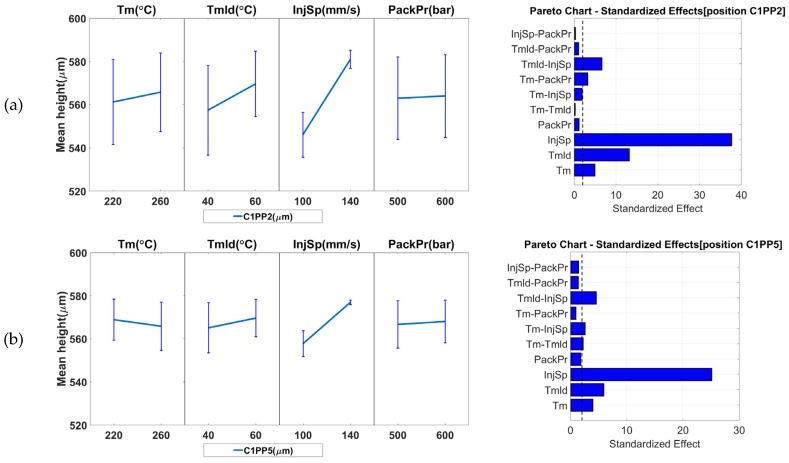
Influence of the IM process parameters on the measurands (pillar height) and possible product fingerprints from cavity: (**a**) C1PP2 (micro pillars far from the gate) and (**b**) C1PP5 (micro pillars near the gate). The figure presents the main effects. The error bars in the main effects plots represent the standard deviations from the respective measurand.

**Figure 13:**
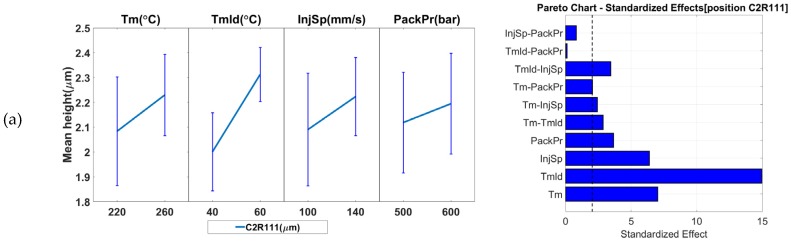

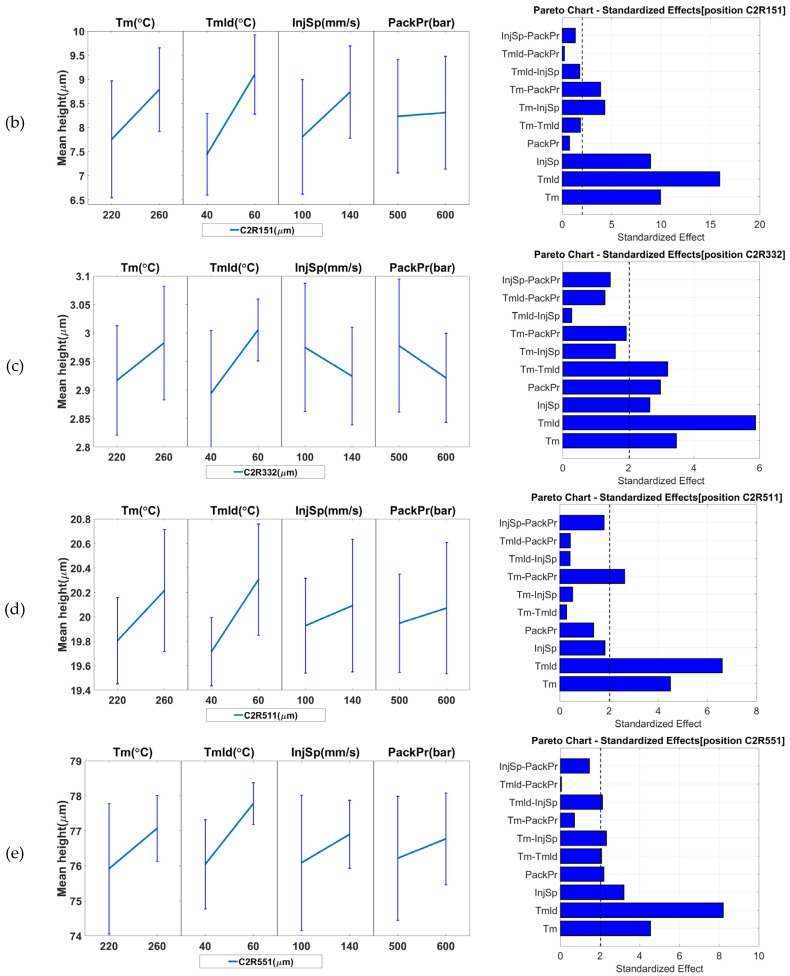
Influence of IM process parameters on the five measurands (micro ridges height—R_height_) and possible product fingerprints from Cavity 2: (**a**) C2R111, (**b**) C2R151, (**c**) C2R332, (**d**) C2R511, and (**e**) C2R551. The figure presents both the main effects (left column) and the Pareto graphs (right column) of standardized effects. The error bars in the main effect plots represent the standard deviations from the respective measurand. The black dashed line in the Pareto plots represents the significance level at a 95% confidence level.

To assess the suitability of the seven measurands as product fingerprints, the results of the measurement sensitivity analysis were evaluated and are reported in Figure 12 and Figure 13. The figures present the main effect plots and the Pareto plots of the standardized effects. Both types of plots reveal the significance that each of the process parameter has on the response and allows identification of the parameters with the highest significance. The error bars in the main effect plots represent the standard deviations of the respective measurand. Such error bars provide a measure of the process’s variation and should be taken into consideration when evaluating the effects of process variations on a measurand. The effects whose variation due to process parameter changes is smaller than the error bar cannot be considered significant and their influence on the measurand is likely to be small or even negligible.

Figure 12a reports the results for the micro-pillar height in position C1PP2 (far from the gate). From the plots, it is evident that the parameter with the greatest influence is the injection speed (*InjSp*). Its increase leads to a 34.9 μm ± 4.2 μm increase in micro-pillar height. In addition, the error bars at the two parameter levels do not overlap. The same can be seen from the Pareto chart. Therefore, the effect of *InjSp* is significant. Similarly, the results for the pillar height in position C1PP5 (near the gate) are illustrated in Figure 12b. From the effect plots, it is evident that the parameter with the greatest influence on the response is the injection speed (*InjSp*) for which an increase of 19.2 μm was observed for the pillar height when increasing *InjSp*. Its effect can be considered significant since the error bars at the two parameter levels do not overlap. Similar conclusions can also be drawn from the Pareto chart as well. In both cases of C1PP2 and C1PP5, only the melt temperature (*Tm*) and the mold temperature (*Tmld*) appears to have an influence among the remaining parameters. However, the error bars at the parameter level do overlap for both parameters, which indicates that the parameters cannot be considered significant.

When the micro features at the two positions are compared, it becomes evident that C1PP2 (far from the gate) is more sensitive to process variations particularly for the increase in *InjSp* and *Tmld*, which results in a larger increase in pillar height at position C1PP2 when compared to position C1PP5 since it is located at the end of the flow path. The reason lies in the rheological behavior of polymers. When higher *Tmld* or *InjSp* is used, the melt viscosity in the cavity is reduced due to either the higher temperature or the shear thinning effect. A lower viscosity level increases the replication fidelity of the micro features, which is beneficial especially for features with a high aspect ratio (2.4-3). In both cases of C1PP2 and C1PP5, the interaction of *Tmld* and *InjSp* appears to have an influence on the responses and is of particular importance for all measurands in Cavity 2. The parameter *Tmld* was the parameter with the largest influence.

Figure 13a shows the results from the R structure (micro-ridge arrays) height measurements in position C2R111 (far from the gate on the right side). From the effect plots, it can be seen that the parameter with the greatest influence on the response was the mold temperature (*Tmld*). Its increase caused a 0.3 μm increase in height. The same conclusion can be drawn from the Pareto chart. The error bars at the two parameter levels do not overlap and, thus, the effect is considered significant. The rest of the parameters all appear to have had an influence as did the 2-way interaction of *Tmld* and *InjSp*. However, the error bars at the parameter levels of the rest of the parameter effects do overlap, which indicates that the parameters cannot be considered significant.

Figure 13b shows the results for the R structure height measurement in position C2R151 (far from the gate on the left side). The results are similar to what was obtained at position C2R111 where the main effect plots indicate that the parameter with the greatest influence on the response was the mold temperature (*Tmld*). Its increase from 40 °C to 60 °C caused a 2.3 μm increase in feature height. For the rest of the parameters, only the packing pressure (*PackPr*) does not have an influence. However, none can be seen as significant as the error bars in the main effect plot overlap for the two parameter levels.

In comparison to positions C2R111 and C2R151 that are located closer to the end of Cavity 2, the features in positions C2R332 (central position, Figure 13c), C2R511 (near the gate on the right, Figure 13d), and C2R551 (left side, Figure 13d) were less sensitive to process variation than the two previously discussed positions. In the case of C2R511 and C2R551 (Figure 13d), an increase in *Tm, Tmld, InjSp*, and *PackPr* parameters resulted in a feature height increase of 0.6 μm and 2.1 μm, respectively, which is within the limit of the error bars. This indicated that none of the parameters may be considered significant. Similarly, for the results of the feature height from position C2R332 (Figure 13c), none of the parameters can be considered significant. However, the increase of *Tmld* caused an increase of 0.11 μm in the feature height while an increase in the parameters of *InjSp* from 100 mm/s to 140 mm/s and *PackPr* from 600 bar to 700 bar had the opposite effect. The reason for this behavior lies in a reduction of the melt viscosity. The combination with the geometry of the shallow (3.8 μm) micro-ridges feature and the orientation of the slope perpendicular to the flow path meant that the polymer at higher injection speeds could pass over the features and a frozen surface layer forms before filling the features. When *PackPr* is considered, in such a shallow feature, the already formed frozen layer cannot be deformed by the higher packing pressure to fill the sharp corners at the bottom of the μ-ridges. In all positions of Cavity 2, the parameter with the largest influence was the mold temperature (*Tmld*), which was followed by the melt temperature (*Tm*). From the Pareto charts, it is evident that the two-way interactions existed mainly at the positions further from the gate where the responses were more sensitive to process variations. This behavior originates from the viscosity changes, which were caused by a change in a combination of parameters. As the pressure dropped along the flow path, the effects of viscosity changed and became more prominent further from the gate. The features far from the gate in both cavities (C1PP2, C2R111, and C2R151) are, therefore, considered more suitable options for product fingerprints.

The analysis of the effects of the different IM process parameters on the seven measurands has provided some indication of the most suitable product fingerprints with respect to their sensitivity to process variations. However, a product fingerprint is required to have a high level of correlation with the overall part quality assessed by a measurand. In the current study, the μ-pillars in position C1PP2 were considered representative for the overall quality of the molded part due to their location close to the end of the flow path and are, thus, regarded as a suitable product fingerprint. A correlation analysis was carried out to determine the most suitable product fingerprints from the feature in Cavity 2. For this purpose, the Pearson correlation coefficient ρ was calculated [22].

**Figure 14:**
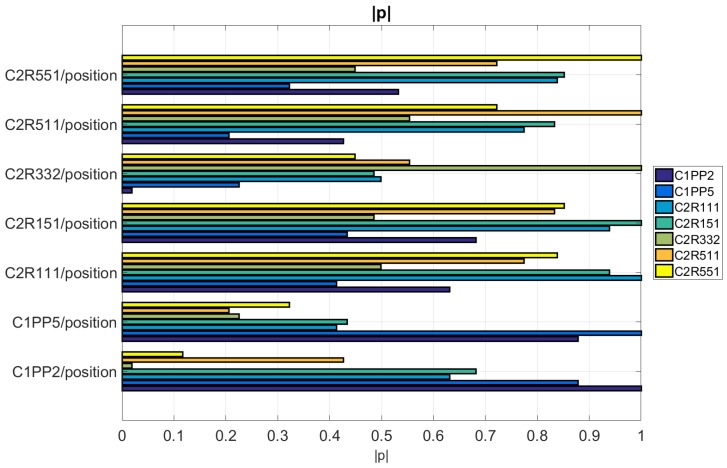
Values of the absolute Pearson correlation coefficient |ρ| calculated from each possible combination of the seven measurands from Cavity 1 and 2. A perfect correlation (|ρ|=1) exists only for combinations of the same dataset.

The calculated |ρ| values for the 49 dataset combinations are shown in Figure 14. It can be seen that the strongest correlations are found for the combinations C2R151/C2R111 (|ρ| = 0.93) in Cavity 2 and C1PP2/C1PP5 (|ρ| = 0.877) in Cavity 1 while the best correlation coefficients between datasets from both cavities were C1PP2/C2R111 (|ρ| = 0.63) and C1PP2/C2R151 (|ρ| = 0.68). The rest of the correlation coefficients varied between |ρ| = 0.43 for measurand combinations within the same cavity and |ρ| = 0.11 for measurand combinations from both cavities.

### 3.2. Process Fingerprint Analysis

To compare process quality indicators, a single value was extracted at each cycle either from the molding machine or from the sensors. Then, a similar analysis explained in the previous section was carried out to identify the most suitable process fingerprint, which must have high process sensitivity and be strongly correlated with the physical measurands (i.e., product fingerprints). In this case, the main effect’s error bars were calculated from the standard error originating from the datasets associated with a particular level of a single parameter. The process fingerprint candidates (35 candidates) described in Figure 12 were calculated for all seven positions (two positions in Cavity 1: C1PP2 and C1PP5 and five positions in Cavity 2: C2R111, C2R151, C2R332, C2R511, C2R551) yielding a total of 245 combinations of process fingerprint and measurands.

**Figure 15:**
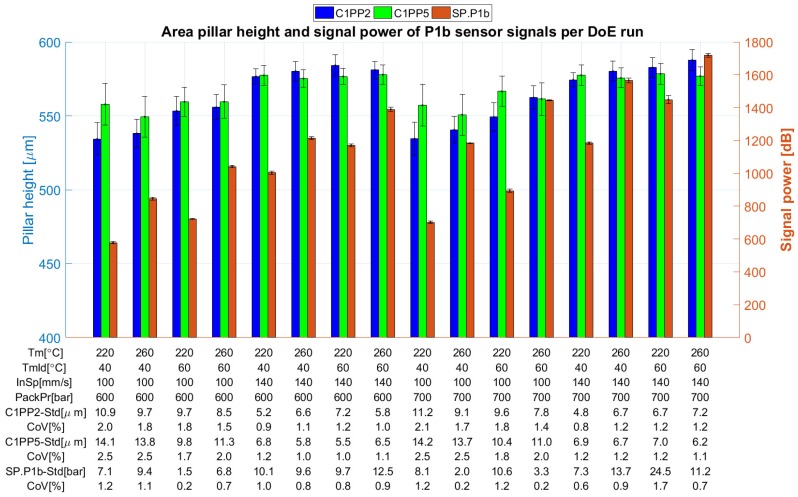
Values of the measurands in positions C1PP2 (far from the gate), C1PP5 (near the gate), and the average power of the signal originating from the piezoelectric pressure sensor *P1b* far from the gate in Cavity 1.

In an initial analysis, the calculated values of the possible process fingerprints were derived by plotting the data and assessing the similarity of the trend in the data, as shown in Figure 15. In most cases, no clearly visible trend exists between the measurement of a measurand position and the calculated process fingerprint candidates. In the case of the integral and signal power of the signals originating from sensor P1b, it is evident that they followed a similar trend to the measurement data from positions C2PP2 and C2PP5 (Figure 15). This preliminary analysis provided an indication of the existence of two combinations of highly correlated datasets (C1PP2 and C1PP5 with *I-P1b* and *SP-P1b*).

**Figure 16:**
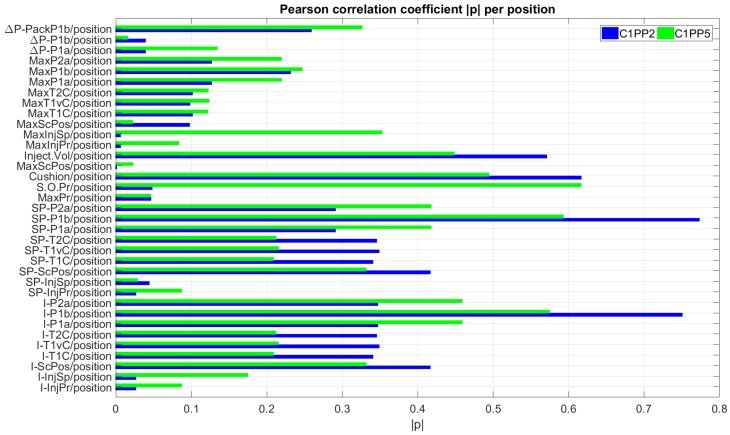
Values of the absolute Pearson correlation coefficient |ρ| calculated from each process fingerprint candidate with respect to the feature height in positions C1PP2 (far from the gate) and C1PP5 (near the gate) of Cavity 1. The process fingerprint candidates are listed in the y axis of the figure.

The analysis continued with a correlation analysis and screening of the 35 process fingerprint candidates coupled with the height data from the seven measurement positions in the molded components. The process fingerprint candidates were separated into five categories described in Figure 11. Figure 16 illustrates the results of the analysis for the process fingerprint candidates with respect to positions C1PP2 and C1PP5. The candidates that originated from the pressure drop values *ΔP-X_i_*/position in correlation with any measurand position have a maximum correlation coefficient of |ρ| = 0.32 for the combination *ΔP-PackP1b*/C1PP5 (*ΔP-PackP1b* denotes the pressure drop from the nozzle to the location of sensors P1b in the cavity considering both the injection and packing phases). They are, therefore, not considered viable for process fingerprints.

The next category of process fingerprint candidates included the maximum values from the signals that were originally obtained from the IM machine and in-mold sensors. The maximum correlation coefficient for the second category of **Max-X_i_** was |ρ| = 0.354 for the combination *MaxT1C*/C2R551,” which denotes the maximum values from the signals from the temperature sensor *T1C* in Cavity 1. The results are similar for signals from sensors *T1vC* and *T2C*. However, due to the low correlation of candidates **Max-X_i_** with the seven measurands, it indicates that they cannot be considered suitable candidates for process fingerprints.

In addition to the calculated maximum values from the signals, the IM machine’s on-board quality control system (i.e., machine data generated, **MD**) provided a number of variables as part of the quality monitoring. Among those variables, the most important ones were: *Max Pressure* (*MaxPr*), *Switch Over Pressure* (*SOPr*), and *Cushion*. *Cushion* is expressed in millimeters and represents the material volume left in the barrel at the end of the cycle. From *Cushion*, in combination with the maximum screw position calculated from the signal *Screw Position*, the injected material volume (*VolInj*) can be derived. This is only an approximate value since the compressibility of the molten material is not taken into consideration in this calculation. The injected volume (*VolInj*) had a maximum correlation of |ρ| = 0.57 to the dataset from position C1PP2. The remaining correlation coefficients for the datasets from all other positions is smaller than 0.44. Similarly, *Cushion* had a maximum correlation coefficient of |ρ| = 0.61 with the dataset from position C1PP2 and can, thus, be considered a suitable process fingerprint candidate. Another suitable candidate process fingerprint was the switch over pressure (*SOPr*), which had a maximum correlation coefficient of |ρ| = 0.61 with the dataset from position C1PP5. The height of the features in position C1PP5 was sensitive to changes in *SOPr* since the change of the packing pressure influenced the replication quality of the pillars in this position and can, thus, be considered a suitable process fingerprint. These candidates are particularly interesting since they are directly provided by the IM machine’s controller and do not require any additional sensor system, which means they allow process monitoring and control to take place at a minimum additional cost.

The fourth category of potential process fingerprints included the integrals of the recorded signals from both machine and in-mold sensor signals. For pressure signals, the integral directly relates to the energy stored in the polymer from the melting, compression, and injection processes taking place in the mold cavity. The largest correlation coefficients of integral levels to the datasets originated from the signals from the piezoelectric pressure sensor *P1b* and the signals from the temperature sensors (*T1C*, *T1vC*, and *T2C*). However, the values from the temperature sensors were taken into consideration since they are greatly dependent on the mold cavity temperature *Tmld*. Their measurement output is biased, so the focus was set on the pressure *P1b* signals. The maximum correlation coefficient of |ρ| = 0.758 was calculated for the combination *I-P1b*/C2R151, which was followed by *I-P1b*/C1PP2 (|ρ| = 0.749), *I-P1b*/C2R111 (|ρ| = 0.749), *I-P1b*/C2R551 (|ρ| = 0.658), and *I-P1b*/C2R511 (|ρ| = 0.655), which suggests that the *I-P1b* values showed good correlation with all datasets with the exception of dataset C2R332. This dataset contained data from the second nominally smallest micro-ridges of the R structure array and was similar (5° inclination—50 μm inclined plane length) to those in position C2R111. Due to the size of the ridges and their position in the center of the molded part (C2R332), the replication fidelity of the micro features did not deviate significantly between the different experimental treatments in comparison to readings from the pressure sensors *P1b* (at the end of the flow path). Therefore, the correlation of datasets *I-P1b* and C2R322 was very weak.

The fifth category of process fingerprint candidates consisted of the power of the signals calculated from the signal inputs. Similarly to the integral values, the largest correlation coefficients of signal power levels with the datasets occurred for the signals from the piezoelectric pressure sensor *P1b* with a maximum correlation coefficient of |ρ| = 0.772 calculated for the combination *SP-P1b*/C1PP2.

From the correlation analysis, a number of promising process fingerprint candidates were selected. The *Cushion*, *VolInj,* and *SOPr* variables are directly provided by the controller on the IM machine. However, the *I-P1b* and *SP-P1b* process fingerprints were found to have an even higher correlation with the overall part quality, which indicates that the positioning of a pressure sensor at the end of the flow path could actively provide valuable data for fast quality assurance and for a warning system for the detection of production quality issues. However, in order to consider these variables as suitable process fingerprints, the variable must be affected by process variation.

**Figure 17:**
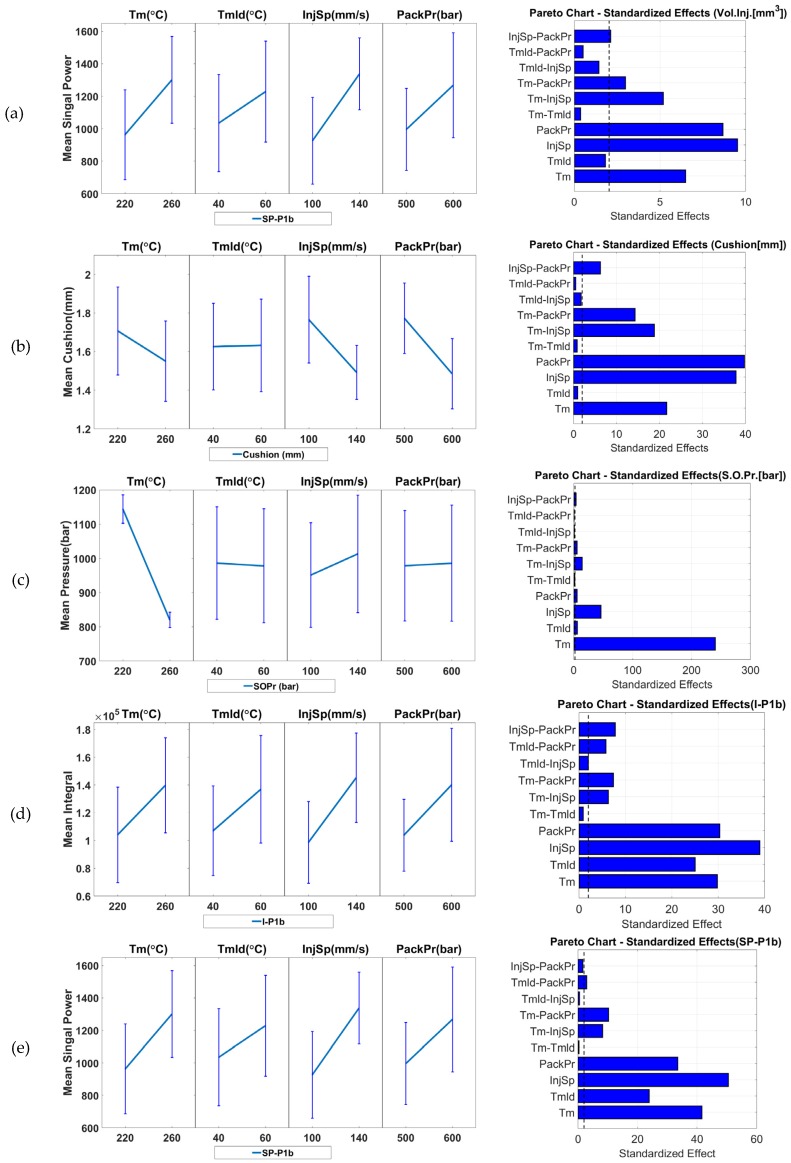
Influence of IM process on the five major process fingerprints candidates: (**a**) *VolInj*, (**b**) *Cushion*, (**c**) *SOPr*, (**d**) *I-P1b*, and (**e**) *SP-P1b*. The figure presents both the main effects (left column) and the Pareto graphs (right column) of standardized effects. The error bars in the main effects plots represent the standard deviations from the dataset of the respective process fingerprint. The black dashed line in the Pareto plots represents the significance level at a 95% confidence level.

To verify the existence of such effects, a process sensibility analysis was carried out for the most prominent candidates. The results are shown in Figure 17. As in the analysis of the product fingerprints discussed in Section 3.1, Figure 17 shows the main effect plots and the Pareto plots of the standardized effects. Both plot types represent the significance that each of the process parameters have on the responses. They make it possible to identify the parameter with the highest significance. As stated for the product fingerprint, such a measure of process variation should be taken into consideration when evaluating the effects of process variation on a measurand.

Figure 17a shows the results for the response of *VolInj* on changes in process parameters. The main effect plots show that the parameter with the greatest influence on the response is the injection pressure (*InjSp*) whose increase from 100 mm/s to 140 mm/s resulted in an increase of 71 mm^3^ in the volume injected (*VolInj*), which was followed by packing pressure (*PackPr*) and the mold temperature (*Tmld*). However, none of these can be considered significant since the error bars at their two levels overlapped. The Pareto chart in the right column shows the significant two-way interactions between *Tm*, *InjSp*, and *PackPr*. Such interactions are expected as the higher level of *Tm* in combination with high levels of either *InjSp* or *PackPr*, reduces the viscosity of the molten polymer, and forces a larger melt volume into the mold cavity. As stated earlier in Section 2.5 and Section 3.2, the values of the *VolInj* response were calculated by using the *Cushion* measurements and, as such, there is a direct dependency with *Cushion* levels. A similar but opposite effect can be seen in Figure 17b for the cushion due to the influence of process variation.

Figure 17c shows the results for the response of the switch over pressure *SOPr* as influenced by the process parameter deviation. From the effect plots, it can be seen that changes in melt temperature can have a major influence on the *SOPr*. An increase of *Tm* from 220 °C to 260 °C resulted in a pressure drop of 61.7 bar so the influence of *Tm* must be considered significant.

The other two most prominent process fingerprint candidates were not derived directly from the IM machine but were recorded with the use of an in-mold cavity pressure sensor. Both the integral and power of the signal were dependent on the amplitude and shape of the signal. The integral of the *P1b* signal (Figure 17d) was mostly influenced by an increase in *InjSp* from 100 mm/s to 140 mm/s. This change caused a drop in the amplitude of the pressure signal and delayed the pressure peak. An increase in *PackPr* from 600 bar to 700 bar increased the plateau amplitude for the packing pressure level and, thus, increased the integral of the signal since it was calculated for both injection and packing phases of the process. The signal power and its integral are both measures of the energy stored in the polymer during the process. Similarly to *I-P1b*, the *SP-P1b* process fingerprint was sensitive to process variations. Considering the correlation coefficients, both indicators may be considered suitable for serving as process fingerprints.

## 4. Conclusions

The present article reports a detailed investigation of the validity of using product and process fingerprints as a new process monitoring concept for fast quality assurance of injection molded components, which adds functional micro features as possible product and process fingerprints. A screening of seven product and 35 process fingerprint candidates was carried out in order to select the most suitable fingerprints based on their sensitivity to process variation and correlation to micro feature replication quality. Topographic analysis of the micro features was conducted off-line by laser scanning confocal microscopy. The signals from machine and external sensors were used for in-line process characterization. Summarizing the previously discussed results, a number of conclusions can be drawn.

- The variation of the IM process parameter settings had an effect on the overall quality and replication of the molded micro-featured surfaces. In particular, the variation affects the quality of the functional micro pillars of the F structure that was positioned in Cavity 1.
- Based on the variation of the injection molding process, the quality of the seven molded structures was assessed and their suitability as a product fingerprint was verified in a correlation analysis of combinations of measurands. For Cavity 1, the dataset C1PP5 (micro pillars near the gate) could be used to predict the quality of the dataset from position C1PP2 (micro pillars far from the gate) since the correlation C1PP2/C1PP5 was |ρ| = 0.877. For Cavity 2, the results show that the dataset C2R111 (micro ridges far from the gate) can be used to predict the quality of the dataset from position C2R151 since the correlation C2R151/C2R111 was |ρ| = 0.93.
- Additionally, when looking for a product fingerprint in one cavity that could also be representative of the replication in the other cavity, the quality of features in position C1PP2 (micro pillars far from the gate) can be produced by the dataset from position/measurand C2R111 or C2R151 with a correlation C1PP2/C2R111 of |ρ| = 0.63 and C1PP2/C2R151 (|ρ| = 0.68). This observation suggests that feature C2R151 (micro ridges far from the gate) was the most suitable product fingerprint for both cavities and can be placed on μ-pillar structured components far from the position of the gate in order to be used for the monitoring of the microstructures’ quality.
- From the wide variety of process fingerprint candidates assessed for their suitability for monitoring the quality of microstructure replication, a small number of candidates proved to be suitable when considered in combination with specific product measurands. These process fingerprints originated from two data sources including one from the in-mold sensors and the other from the IM machine itself.
- It was concluded that the functional features at position C1PP2 can be successfully monitored with the use of two process fingerprints such as *SP-P1b* (power of signals “**SP**” originated from pressure sensor *P1b*, located far from the gate at the end of the flow path, correlation |ρ| = 0.772) and I-P1b (integral “*I*” of signals originated from pressure sensor *P1b*, correlation |ρ| = 0.749). These fingerprints are also suitable for the monitoring of a micro feature quality in positions C2R111 (|ρ| = 0.749), C2R151 (|ρ| = 0.758), C2R511 (|ρ| = 0.655), and C2R551 (|ρ| = 0.658). Process fingerprints *SP-P1b* and *I-P1b* are more suitable for monitoring the features at positions located closer to the end of the flow path. In comparison, the micro structures in Cavity 2 cannot be monitored reliably by process fingerprints derived from machine data while the larger micro structures in Cavity 1 can.
- The machine process parameter *Cushion* is a suitable process fingerprint and can be used to monitor the quality of micro pillars in position C1PP2 (correlation |ρ| = 0.61). *SOPr* is also a suitable process fingerprint and can be used to monitor the quality of micro pillars in position C1PP5 (| ρ| = 0.61).
- In summary, *SP-P1b*, *I-P1b*, *Cushion*, and *SOPr* are suitable process fingerprints for the monitoring of the functional micro pillars in positions C1PP2 and C1PP5. The *I-P1b* and *SP-P1b* process fingerprints provide higher correlation to the overall part quality and indicate that the positioning of a pressure sensor at the end of the flow path can actively provide data for fast quality assurance, which renders high intensity dimensional metrology efforts unnecessary.

Based on the discussed research work, the presented product and process fingerprint concept could be applied to injection molded components with micro-structured geometries similar to the test disk geometries used for this experiment such as plates. Therefore, future work will attempt to validate the concept for different molded components with functional microstructures and to assess the robustness of product and process fingerprint performance in long production runs while implementing mathematical models to predict the quality of the manufactured components.

## Figures and Tables

**Table 1:** Product feature characteristics and sensor outputs.

**Product Features**						
**Name**	**Cavity #**	**Type**	**Position from Gate/Lateral**	**Characteristics**		
				**Top Diameter**	**Bottom Diameter**	**Pillar Height (P_Height_)**
C1PP2	1	Micro pillars	Far	Ø200 μm	Ø250 μm	600 μm
C1PP5	1	Micro pillars	Near	Ø200 μm	Ø250 μm	600 μm
-	-	-	-	Inclination	Incline plane length	R_height_
C2R111	2	Micro ridges	Far/right side	5°	50 μm	2.7 μm
C2R151	2	Micro ridges	Far/left side	20°	50 μm	11.8 μm
C2R332	2	Micro ridges	Middle	5°	50 μm	3.8 μm
C2R511	2	Micro ridges	Near/Right	5°	250 μm	21.6 μm
C2R551	2	Micro ridges	Near/Left	20°	250 μm	81.34 μm
**Sensors**						
**Name**	**Cavity #**	**Type**	**Position from Gate**	**Output**		
P1a	1	Piezoelectric	Near	Transient pressure		
P1b	1	Piezoelectric	Far	Transient pressure		
P2a	2	Piezoelectric	Near	Transient pressure		
T1C	1	Group N	Far	Transient temperature		
T1vC	1	Group N	In the mold block behind Cavity1	Transient temperature		
T2C	2	Group N	Far	Transient temperature		

**Table 2:** In-mold piezoelectric sensor sensitivity.

Piezoelectric Sensor Sensitivity			
Piezoelectric Sensor	Channel	Sensitivity	Unit
P1a	P2	1.860	pC/bar
P1b	P3	1.880	pC/bar
P2a	P4	1.900	pC/bar

**Table 3:** Experimental process parameters—Full factorial DOE.

Parameter	Symbol	Unit	Low Level	High Level
Melt temperature	*Tm*	°C	220	260
Mold temperature	*Tmld*	°C	40	60
Injection speed	*InjSp*	mm/s	100	140
Packing pressure	*PackPr*	bar	600	700

**Table 4:** CLSM measurement settings used for μ-pillars (F structure) and μ-ridges (R structure).

CLSM Measurement Settings		
Parameter	μ-Pillars (F Structure)	μ-Ridges (R Structure)
Objective	50×	50×
Aperture N	0.55	0.95
Lens Working Distance	8.7 mm	0.35 mm
Measurement Range	620 μm	1200 × 200 μm
Brightness	7220	7220
Z pitch	0.36 μm	0.2 μm

**Table 5:** List of recorded variables.

Machine Data (Single Value)	Machine Signals	In-Mold Sensor Signals
Injection time (s)	Injection/Pack. Pressure (bar)	T1C (Cavity1) (°C)
Max Pressure (bar)	Injection speed (mm/s)	T1viaC (Cavity1) (°C)
Switch Over Pressure (bar)	Screw position (mm)	T2C (Cavity2) (°C)
Cushion (mm)		P1a (Cavity1-gate) (bar)
Dosage time (s)		P1b (Cavity1-end) (bar)
Cycle time (s)		P2a (Cavity2-gate) (bar)
